# Temporal bone radiology report classification using open source machine learning and natural langue processing libraries

**DOI:** 10.1186/s12911-016-0306-3

**Published:** 2016-06-06

**Authors:** Aaron J. Masino, Robert W. Grundmeier, Jeffrey W. Pennington, John A. Germiller, E. Bryan Crenshaw

**Affiliations:** Department of Biomedical and Health Informatics, The Children’s Hospital of Philadelphia, 3535 Market Street, Suite 1024, Philadelphia, PA 19104 USA; Department of Pediatrics, Perelman School of Medicine at the University of Pennsylvania, 34th Street & Civic Center Boulevard, Philadelphia, PA 19104 USA; Center for Childhood Communication, The Children’s Hospital of Philadelphia, 34th Street & Civic Center Boulevard, Philadelphia, PA 19104 USA; Department of Otorhinolaryngology: Head and Neck Surgery, Perelman School of Medicine at the University of Pennsylvania, 3400 Spruce Street, Philadelphia, PA 19104 USA

**Keywords:** Human-in-the-loop, Machine learning, Natural language processing, Radiology, Audiology

## Abstract

**Background:**

Radiology reports are a rich resource for biomedical research. Prior to utilization, trained experts must manually review reports to identify discrete outcomes. The Audiological and Genetic Database (AudGenDB) is a public, de-identified research database that contains over 16,000 radiology reports. Because the reports are unlabeled, it is difficult to select those with specific abnormalities. We implemented a classification pipeline using a human-in-the-loop machine learning approach and open source libraries to label the reports with one or more of four abnormality region labels: inner, middle, outer, and mastoid, indicating the presence of an abnormality in the specified ear region.

**Methods:**

Trained abstractors labeled radiology reports taken from AudGenDB to form a gold standard. These were split into training (80 %) and test (20 %) sets. We applied open source libraries to normalize and convert every report to an n-gram feature vector. We trained logistic regression, support vector machine (linear and Gaussian), decision tree, random forest, and naïve Bayes models for each ear region. The models were evaluated on the hold-out test set.

**Results:**

Our gold-standard data set contained 726 reports. The best classifiers were linear support vector machine for inner and outer ear, logistic regression for middle ear, and decision tree for mastoid. Classifier test set accuracy was 90 %, 90 %, 93 %, and 82 % for the inner, middle, outer and mastoid regions, respectively. The logistic regression method was very consistent, achieving accuracy scores within 2.75 % of the best classifier across regions and a receiver operator characteristic area under the curve of 0.92 or greater across all regions.

**Conclusions:**

Our results indicate that the applied methods achieve accuracy scores sufficient to support our objective of extracting discrete features from radiology reports to enhance cohort identification in AudGenDB. The models described here are available in several free, open source libraries that make them more accessible and simplify their utilization as demonstrated in this work. We additionally implemented the models as a web service that accepts radiology report text in an HTTP request and provides the predicted region labels. This service has been used to label the reports in AudGenDB and is freely available.

**Electronic supplementary material:**

The online version of this article (doi:10.1186/s12911-016-0306-3) contains supplementary material, which is available to authorized users.

## Background

Electronic health records (EHRs) contain significant amounts of unstructured text that pose a challenge to their secondary use as a research data source [1, 2]. Prior to research utilization, EHR text data, such as physician notes and radiology reports typically must be converted to discrete values, e.g. outcome labels. In the absence of automated processing, this requires trained data abstractors to manually review the text sources and identify discrete values of interest. Such manual review may be time consuming and expensive, particularly for large data sets. Natural language processing (NLP) and machine learning (ML) methods present an alternative to manual text review. These methods have been applied to automate EHR text analysis in a variety of studies including phenotype extraction, adverse drug-event identification, and domain-specific radiology report classification [3–8].

In audiologic and otologic research, the ability to use anatomic information described in radiology is essential to understand the causes of hearing loss for research subjects and to develop new treatment modalities [9–12]. The Audiological and Genetic Database (AudGenDB), a public, de-identified observational research database derived from EHR data sources, contains over 16,000 de-identified, unlabeled radiologist reports [13]. Because the reports are unlabeled, it is difficult for researchers to select reports that contain abnormalities in a specific region, e.g. the inner ear. Two straightforward methods to be considered are keyword searches and International Classification of Diseases (ICD9) based searches [14]. As shown in the work presented here, these approaches lack sensitivity (recall) for this data set, and thus fail to identify most of the reports that contain an abnormality. Therefore, to facilitate the effective use of anatomic information contained in radiology reports for audiology research, we adopted a machine learning procedure.

Ideally, we would like to utilize a fully automated knowledge extraction system for which it would be necessary only to supply radiology reports. The system would generate labels that correspond to entities (e.g. vestibular aqueduct) and attributes (e.g. enlarged) identified in each report to be used for search indexing. Although significant progress has been made in the biomedical domain toward the development of such systems for text analysis [15, 16], fine-tuning is usually necessary to achieve acceptable performance for specific use cases. This requires training documents to be labeled with detailed concepts from the standardized ontology utilized by the system (e.g. UMLS [17]). The size and complexity of such ontologies places a potentially prohibitive burden on the labeler, typically a physician or study staff member, who is required to learn at least part of the ontology in order to perform the annotation task. Additionally, the granularity of the ontology may be inappropriate for the use case. For example, we sought to enable document search for abnormalities in broad ear regions for which highly granular labels are unnecessary. More generally, existing NLP systems [18, 19] can extract pre-specified features (e.g. word tokens, parts of speech tags) from natural language text. However, it remains necessary to determine which features extracted by these systems and which classification models are appropriate for our task. As these steps are likely to benefit from human input, we adopted an approach that utilizes aspects from domain-expert-centered knowledge discovery [20] and human-in-the-loop (HIL) machine learning [21]. In this framework, the domain expert, a physician in our study, plays a central, rather than periphery, role in the knowledge extraction process that includes data selection criteria, document labeling requirements, analysis, and evaluation. The HIL approach enables humans to guide the machine learning process through data, feature and model selection based on expert knowledge and task specific requirements.

Implementing this process, we developed a classification pipeline that uses freely available open-source ML and NLP libraries to label temporal bone radiology reports with one or more of four abnormality region labels: *inner, middle, outer,* and *mastoid,* indicating the presence of an abnormality in the specified ear region. We subsequently incorporated the pipeline into a web service that provides labels for reports submitted via HTTP requests to more broadly enable the audiology research community to effectively use the important anatomic information that is typically buried in radiology reports. Our successful application of these ML and NLP methods to a novel data source, AudGenDB, provides further evidence of their generalizability and that such methods can be effectively utilized in production biomedical software environments.

## Methods

We conducted a retrospective analysis of de-identified radiologist reports obtained through the AudGenDB web query interface. Although AudGenDB now contains data from multiple institutions, radiology reports were only available from The Children’s Hospital of Philadelphia (CHOP) at the time of this study. The CHOP Internal Review Board approved the study under the overall AudGenDB project.

### Document labeling

Each radiology report was reviewed by one of two study staff and annotated with one or more labels indicating ear regions in which structural abnormalities were noted. Following a human-in-the-loop approach, we derived annotation requirements from physician expert guidance [22]. It was determined labels for *inner, middle, and outer* ear, and *mastoid* abnormalities were most appropriate for our task. The criteria for a positive label (indicating presence of an abnormality), developed through expert input, were: *inner* for abnormalities of the cochlea, vestibular aqueduct, vestibular nerves, vestibules, or semicircular canals; *middle* for abnormalities of the tympanic membrane, ossicles, stapes, incus, malleus, or scutum; *outer* for abnormalities of the external auditory canal; and *mastoid* for abnormalities of the mastoid regions. A report was labeled *normal* if no abnormalities were identified. Cohen’s Kappa statistic was calculated on a subset of 50 reports that were labeled by both reviewers to assess inter-rater agreement. The labeled dataset was stratified based on presence or absence of abnormalities in at least one region and randomly split into stratified training and test sets containing 80 and 20 % of the reports, respectively.

### Document normalization and feature vector construction

Prior to the training and analysis, we extracted and normalized the *findings* and *impression* sections from each radiology report. The radiology report sections are consistently demarcated with section headers (e.g. FINDINGS). To extract these sections, we developed a custom Python program that reads each line of the file. It records all lines in the file after the FINDINGS header until the next section header. Similarly, it records all lines after the IMPRESSION header until the next section header. These extracted sections were then normalized. The normalization step uses the Python Natural Language Processing Toolkit (version 2.0.4) and custom regular expression patterns to: replace decimal numbers (e.g. *3.14*) with *number*; replace units (e.g. *years*) with *unit*; remove common English words (except for “no”, “not”, and “under” which were deemed relevant to the specific classification task); and replace all words with their word-stems [19].

After normalization, every report was tokenized (separated into individual words). The tokens obtained from the training set reports were used to create an n-gram (sequence of *n* consecutive words or characters) collection representing all n-grams that occur in at least one *training* report. Note that test reports were also tokenized, but were not used to construct the n-gram collection. This implies that an n-gram that occurs in a test report but not in any training report will not appear in the n-gram collection. Because such an n-gram does not appear in the training set, it would provide no information during the training process and consequently not contribute to the classification model. After tokenization, we converted each report to a numerical feature vector (FV) using the Python *scikit-learn* library (version 0.16.1, with NumPy version 1.10.1) [23]. FVs were constructed from the n-gram features in the report. Rather than adopt a pre-specified feature set or use unsupervised feature learning as would be done in a fully automated machine learning process, we employed a HIL technique and evaluated several potential candidate features identified from expert knowledge. The specific FV configuration was determined separately for each classifier as part of the model selection and evaluation process described below. We considered FVs composed of word only n-grams and character only n-grams with *n* in the range [1, 3]. For both word and character based FVs, we evaluated binary values, where element *i (0 or 1)* indicates the *i*^*th*^ feature (from the complete n-gram collection) is absent or present in the report, and feature counts, where element *i* is the number of times the *i*^*th*^ feature occurred in the report.

### Model construction and evaluation

As with feature selection, we adopted a HIL procedure to select and tune the appropriate classification algorithms, in contrast to a fully automated process that would implement a pre-specified method. We evaluated logistic regression, support vector machine (SVM), decision tree, random forest, and naïve Bayes models. Model construction required hyperparameter selection for the associated learning algorithms. The hyperparameters include model specific parameters (e.g. regularization constant) and the specific FV configuration options (character vs. word N-grams, and binary features vs. counts). Only binary FVs were considered for the naïve Bayes classifier, as that is an algorithm assumption. We used the *scikit-learn* library, which includes implementations of the considered learning algorithms, to perform a grid search and select the optimal hyperparameters for each model. Hyperparameter combinations were evaluated by 5-fold cross validation [24]. For each learning algorithm and ear region, the model hyperparameters and FV combination with the best cross-validation performance was selected and the model was re-trained using the entire training data set. These models were then run for the test set to evaluate generalization error and the best performing one in each ear region was selected for use in the report-labeling pipeline.

### Web service implementation

To make the report classifiers available to applications, specifically AudGenDB, we implemented a web service that provides the predicted labels for each of the four ear regions. The web service is a REST implementation created in Python using the open-source Flask library. The REST service accepts HTTP *POST* requests that include one or more reports to be labeled and responds with the predicted labels.

### Alternative methods

We considered two alternative approaches for comparison to our ML models. First, we created a keyword list for each ear region [see Additional file 1]. We then performed a keyword search where we considered a report *abnormal* for a specific ear region if it contains any region specific keywords. In the second alternative, we identified a list of ICD9 diagnosis codes that pertain to abnormalities of the ear. These were assigned to one or more ear regions based on the code description [see Additional file 1]. For each radiology report, we obtained the ICD9 codes annotated to the associated patient from AudGenDB. A radiology report was considered abnormal for a given ear region if the patient’s diagnosis codes included any of the ICD9 codes assigned to that ear region.

## Results

**Fig. 1:**
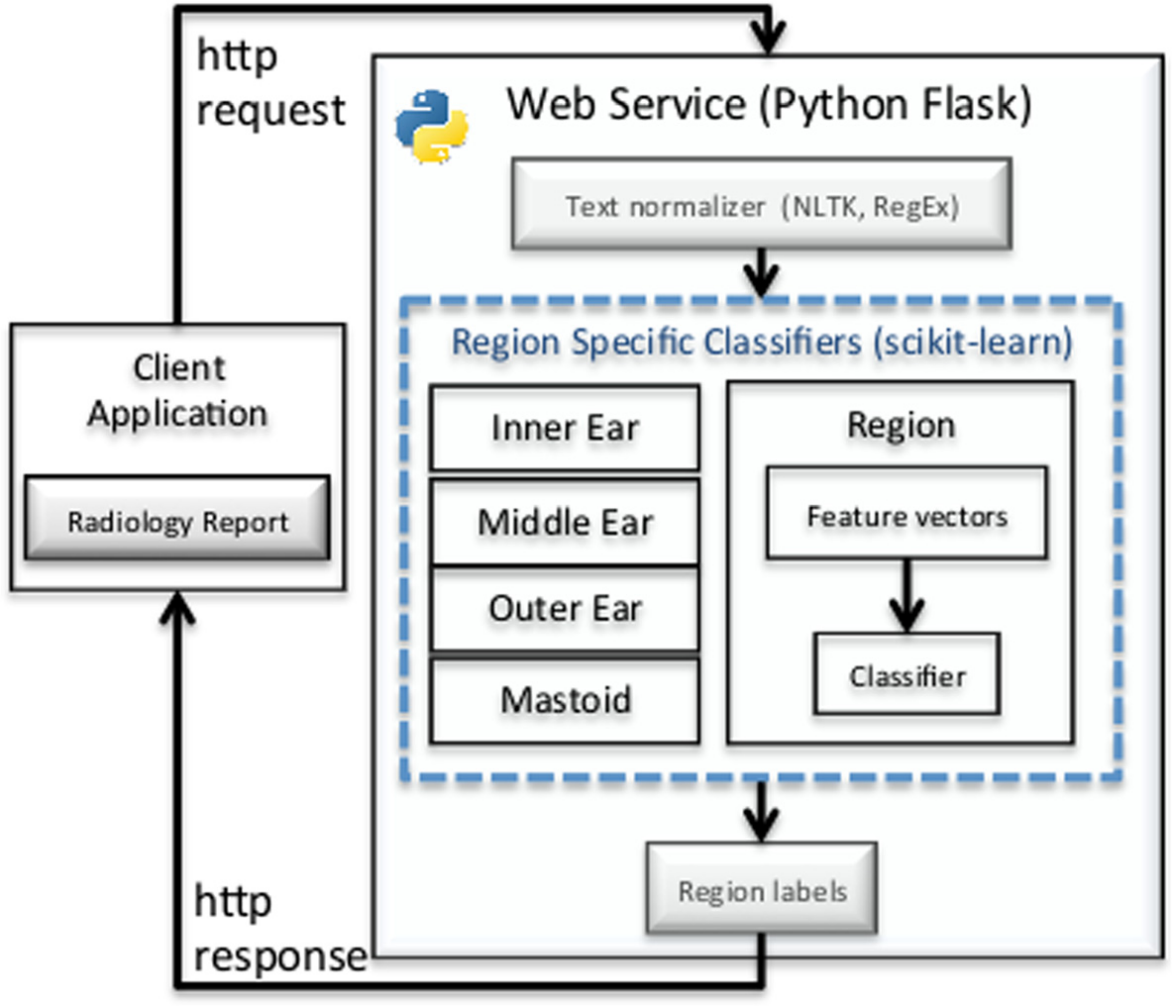
Web service and classification pipeline architecture. Client requests include radiology reports that are first normalized and then classified by four region specific models. Label values are returned to client via an HTTP response

We developed a multi-label classification pipeline, illustrated in Fig. 1, to classify input radiologist reports as *normal* or *abnormal* for each of four ear anatomical regions: *inner, middle, outer* and *mastoid.* Each report is pre-processed to extract the *findings* and *impression* sections and to perform text normalization. The normalized sections are then converted to a discrete numerical feature vector (FV). The FV is input to the four separate machine learning classifiers that label the report *normal* or *abnormal* relative to a specific ear region. The models are made accessible to client applications via a web service.

Our dataset consisted of 726 radiology reports composed primarily of computed tomography (CT) scans of the temporal bones and a small number of Magnetic Resonance Imaging (MRI) scans of the brain. Inter-rater agreement was near perfect (kappa 0.94, 0.90, 0.88, and 0.82 for the *inner, middle, outer, and mastoid* regions, respectively) [25]. Based on these results, we determined that single report assessment by reviewers working in parallel to generate a larger training set was the most efficient resource utilization. A double review with consensus reconciliation of discordant assessment may have yielded marginally better labels, but would have been feasible only for a smaller report set.

The dataset was randomly split into training and test sets consisting of 580 (80 %) and 146 (20 %) reports, respectively. The percentage of training and test set normal and abnormal reports was equal to within 1 % between the two sets, where an abnormal report is one with an abnormality in at least one region. The percentage of training and test set normal and abnormal reports by region was equal to within 6.13 % across the four regions. Table 1 details the training and test set label distributions. The table reflects that while the majority of documents contained at least one abnormality (indicated by the *At least one* region) only a minority fraction contained an abnormality for a given ear region.

**Table 1:** Abnormal annotation distribution

Region	Training set	Test set
At least one	62.41 % (362)	62.33 % (91)
Inner	26.72 % (155)	26.03 % (38)
Middle	37.59 % (218)	40.41 % (59)
Outer	13.79 % (80)	14.38 % (21)
Mastoid	30.86 % (179)	36.99 % (54)

Column values indicate the percentage of documents (values in parenthesis indicate absolute number of documents) that were labeled as abnormal for the given region. A document as a whole was considered abnormal if it contained an abnormality in at least one region. The training and test sets contain a total of 580 and 146 documents, respectively

Cross-validation was used to select model hyperparameters (e.g. regularization constant) and feature vectors (e.g. bigram counts) for each candidate model as described in the methods. Table 2 lists the optimal values for the best classifier for each region among those considered in the grid search. Table 3 details quantitative test set performance metrics for the best region classifiers. For each region, the best model was selected as that with the highest accuracy on the test set. In the event of a tie, the model with the best F1-score was selected. This occurred for both the inner region, where there was an accuracy tie between the logistic regression and the linear SVM models, and for the mastoid region where there was tie between the decision tree and random forest models. It is important to note that the data sets are imbalanced in every ear region with the majority normal class representing a minimum 59.6 % (middle ear) up to a maximum 85.6 % (outer ear) of the test cases (Table 1). In our results, the best model accuracy is greater than the majority class percentage across all regions (range 7.38–30.4 %, Table 3) implying that the models learned criteria that go beyond simply selecting the majority class.

**Table 2:** Best classifier hyperpameters by ear region

	Feature Vector Hyperparameters			
Region	Best classifier	n-gram Range	Word/Character	Model Hyperparameters
Inner Ear	SVM (Linear)	1–2	Word	Cost parameter, C = 0.1
Middle Ear	Logistic Regression	1–3	Word	Regularization cost, l = 0.1
Outer Ear	SVM (Linear)	1–3	Word	Cost parameter, C = 0.333
Mastoid	Decision Tree	1–3	Character	Max depth = 2

**Table 3:** Best classifier test set performance metrics

Region	Inner	Middle	Outer	Mastoid
Accuracy	90 % (+16.0)	90 % (+30.4)	93 % (+7.38)	82 % (+19.0)
F1 Score	0.82	0.85	0.71	0.74
NPV	0.94	0.85	0.93	0.83
PPV	0.82	1.0	0.92	0.80
Sensitivity	0.82	0.75	0.57	0.69
Specificity	0.94	1.0	0.99	0.90

The values in parenthesis in the accuracy row are the percent difference compared to the majority class. NPV is negative predictive value, PPV is positive predictive value. The best classifiers by region were SVM (linear) for the inner and outer ear, logistic regression for the middle ear, and decision tree for the mastoid

To gain further insight into the ML model performance, we consider the test set confusion matrices shown in Table 4 for the best region classifiers. The confusion matrices provide an intuitive performance assessment when we consider the number of misclassified reports (off-diagonal entries) relative to the correctly classified examples (diagonal elements). From the confusion matrices, we observe that the models performed very well on the normal cases as may be expected for the majority class for imbalanced data sets. We also see that the best classifier models incorrectly classify many of the positive test cases, i.e. the minority class, particularly for the outer ear and mastoid regions. This is reflected by the sensitivity results (range 0.57–0.82) shown in Table 3. The nature of these errors can be understood by considering the presence of bias and variance in the training process. Bias occurs, independent of training set size, when the model and its associated assumptions are such that there is a non-zero expected error. Variance occurs due to model sensitivity to fluctuations in the data set and is dependent on training set size. We can observe the impact of these two error sources by examining the learning curves for the best classifiers. A learning curve graph illustrates a selected model performance metric on the training and validation sets as a function of the number of training examples. In the ideal scenario where there is low bias and low variance, we expect the training and validation curves to approach the maximum metric value. In the presence of bias alone, we expect the training curve and the validation curve to both approach a limiting value that is less than the maximum. In the presence of variance alone, we expect the training curve to approach the maximum metric value. However, while the validation curve will increase smoothly with the number of training examples, it will fail to achieve the maximum value. Naturally, most practical cases involve both error sources in which case the training curve will approach a value that is greater than the validation curve but less than the maximum metric value.

**Table 4:** Best classifier confusion matrices by ear region

Inner Ear: SVM Linear Kernel			Middle Ear: Logistic Regression		
	Predicted Label			Predicted Label	
Actual Label	Normal	Abnormal	*Actual Label*	Normal	Abnormal
Normal	101	7	Normal	87	0
Abnormal	7	31	Abnormal	15	44
Outer Ear: SVM Linear Kernel			Mastoid: Decision Tree		
	Predicted Label			Predicted Label	
Actual Label	Normal	Abnormal	*Actual Label*	Normal	Abnormal
Normal	124	1	Normal	83	9
Abnormal	9	12	Abnormal	17	37

Test set confusion matrices for best learning algorithm for each ear region. The confusion matrices provide the true and false counts for normal and abnormal documents as labeled by the classification algorithm

**Fig. 2:**
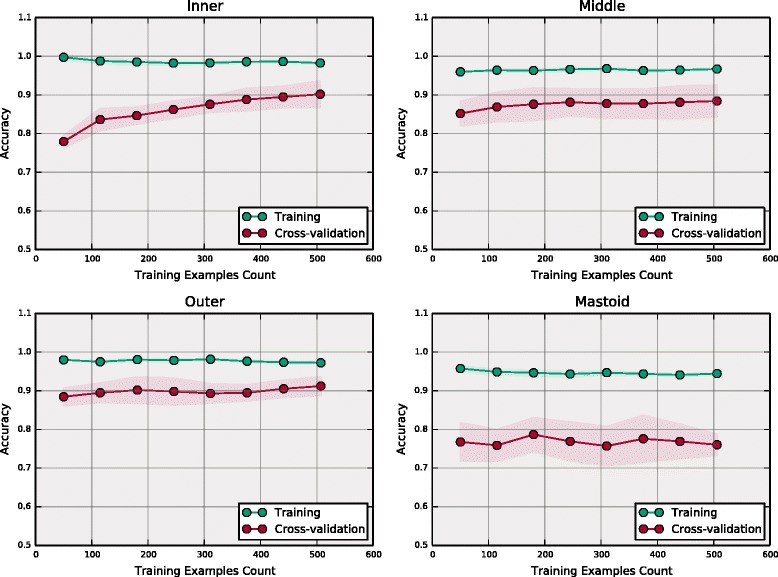
Best classifier learning curves. From top left to bottom right, best classifier model learning curves for the inner ear (linear SVM), middle ear (logistic regression), outer ear (linear SVM), and mastoid (decision tree). The curves show the training and validation accuracy as a function of the training set size. Performance is evaluated by 5-fold cross validation. The green (red) curves indicate performance on the training (cross-validation) report sets. Each data point (circles) is the average accuracy value over the 5 folds. The shaded region indicates the standard deviation

In Fig. 2, we plot the best classifier learning curves for each ear region with accuracy as the performance metric. The inner ear learning curve (Fig. 2, top left) clearly demonstrates the presence of variance. The model achieves nearly 100 % accuracy on the training set whereas the validation curve monotonically increases with additional training examples but fails to reach perfect accuracy. The middle ear learning curve (Fig. 2, top right) indicates both bias and variance are present. The training curve is essentially constant at approximately 97 % accuracy (bias) and the validation curve, while increasing with additional examples, fails to approach the training curve (variance). Similar behavior is observed for the outer ear (Fig. 2, bottom left) and the mastoid (Fig. 2, bottom right). The mastoid learning curve demonstrates the most severe bias, indicated by the fact that the training curve is constant at a relatively low value of 93 %. This model also demonstrated the strongest variance as indicated by the large separation (approximately 15 %) between the training and validation curves.

We considered a keyword search and ICD9 based search as baselines for comparison with the ML models. The accuracy and F1-scores for these alternative methods are shown in Table 5. In all cases, the best machine learning model yields superior accuracy and F1-score values. We assessed the statistical significance of these accuracy differences by McNemar’s test using exact binomial probability calculations [26]. The McNemar’s test statistic for the best ML classifier output labels on the test set relative to those of the ICD9 based model yielded *p*-values less than 0.05 across all regions. This suggests the model outputs are not correlated and that the performance increases observed for the ML classifiers are statistically significant. Similarly, comparing the best ML classifier outputs to the keyword search outputs yielded p-values less than 0.05 for the inner, middle, and mastoid regions. This suggests the performance increase observed for the ML models over the keyword search in those regions is also statistically significant. For the outer region, however, the p-value (0.42) was much greater than 0.05 suggesting that the model outputs are strongly correlated for that region. This is likely due to the strong class imbalance (86 % normal) and small number of positive examples (21) in the outer region for the test set. This situation represents a difficult learning environment for the ML classifier in which it performs poorly on the positive cases. This is reflected by the low sensitivity (0.57) of the best ML classifier for the outer ear region (Table 3).

**Table 5:** Keyword and ICD9 search method performance

	Accuracy			F1-Score		
Region	Best classifier	Keyword	ICD9	Best classifier	Keyword	ICD9
Inner	90 %	75 %	60 %	0.82	0.42	0.60
Middle	90 %	67 %	62 %	0.85	0.67	0.15
Outer	93 %	86 %	86 %	0.71	0.33	0.0
Mastoid	82 %	65 %	68 %	0.73	0.14	0.28

As described in the methods, we extracted the findings and impressions sections of the radiology report and performed text normalization prior to training and testing. As these steps were not part of the cross validation grid search, we examined their impact by evaluating the performance of the *best logistic regression* classifier for each region when separately removing each one of the preprocess steps. The best logistic regression classifier refers to the hyperparameter configuration that yielded the best cross validation results for the logistic regression model for a given region. In the case where extraction of the *findings* and *impression* sections was not performed, i.e. the entire report was used in training and test set evaluation, the test set accuracy difference was less than 2.1 % across all regions. The McNemar test statistic p-value was greater than 0.6 across all regions when comparing the output labels with and without separate extraction of the findings and impression sections. Similarly, we evaluated the best logistic regression classifier when text normalization was not performed. The test set accuracy difference was less than 4.1 % across all regions and the McNemar test statistic p-value was greater than 0.05 across all regions. These observations suggest that these preprocess steps may provide little benefit for this particular data set. We ultimately chose to keep them in our pipeline because they did not hinder performance and they are common practice in similar studies.

**Fig. 3:**
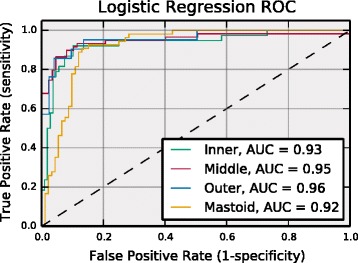
Logistic regression receiver operating characteristic (ROC) by region. The ROC curves for the best performing logistic regression model for each ear region. The dashed line is the expected performance for a random binary classifier. Area Under the Curve (AUC) values closer to 1.0 indicate high performance with low false positive and false negative events

Although the best learning algorithm varied by region, the accuracy difference between the overall best classifier and the best logistic regression classifier was less than 2.75 % across all regions. Furthermore, the McNemar’s statistic as computed for the best classifier output labels and the best logistic regression classifier output labels yielded p-values of 1.0, 0.5, and 0.1 for the inner, outer, and mastoid regions, respectively. For the middle ear region, the best logistic regression classifier was the overall best classifier. These p-values suggest that the best overall model and the best logistic regression model output labels should be considered correlated. Based on these observations, we could reasonably select the logistic regression model for all regions. The logistic regression model is desirable because it can be easily tuned to improve specificity or sensitivity. The Receiver Operating Characteristic (ROC) curves across all regions for the logistic regression model are shown in Fig. 3. An ROC curve illustrates the tradeoff between controlling false positives and negatives through the dependence between sensitivity (or recall) and specificity as a function of the classification decision threshold. The ROC area under the curve (AUC) is a point metric that is often used to assess model performance. For the logistic regression classifier, the AUC is greater than 0.92 across all regions indicating good performance. The ROC curves indicate that this model can simultaneously control false positives and negatives reasonably well for this data set. Further, it can also be tuned to strictly control false positives (negatives) without significantly reducing false negative (positive) control.

## Discussion

Robust methods that reduce the need for manual chart review to identify pertinent radiology reports are critical to support observational clinical studies for hearing loss research. We have demonstrated that standard ML and NLP methods may address this challenge when supported by human expert guidance. These methods significantly outperformed the keyword and ICD9 based search alternatives. Although other baseline methods may be superior, the intent of this work was to demonstrate the effectiveness of ML methods on an audiologic data set as compared to approaches likely to be used by most researchers performing observational clinical studies. Our fundamental criteria for baseline method selection was therefore to rely only on filtering methods and information readily available in the AudGenDB database. We also chose to rely on well-reported ML and NLP methods that are available in free libraries for many programming languages including Python (as we used for our experiment) and R [23, 27]. It is possible that superior results could be obtained through more advanced methods. For instance, the word count vectors used to represent documents could be replaced with word embedding methods such as *word2vec* and *GloVe* that capture additional semantic information [28, 29]*.* More advanced models such as recurrent neural networks that readily account for word order and relations between words would also likely be beneficial [30]. However, we were motivated in part to demonstrate that standard ML and NLP methods could achieve reasonable success on a novel clinical data set.

As described recently [31], the free availability of NLP tools in “out of the box” software packages makes them more accessible to various researchers in numerous settings. However, as the work presented here illustrates, in most cases some form of human guidance is required to develop a successful machine learning application. Accordingly, we adopted concepts from human-in-the-loop machine learning [20, 21] for document annotation, feature selection, and model evaluation. We hypothesize that additional HIL ML concepts could be beneficial to this and similar studies. Specifically, as shown in [20], human knowledge can be integrated into the ML model training process to reduce the necessary training data by starting with a small training set and having the human expert select and label additional examples iteratively based on model performance. This is supported by our results as seen in the learning curves in Fig. 2 that indicate three region classifier models required only a small fraction of the overall training set to achieve maximum performance. This suggests that an iterative HIL approach could have significantly reduced our training data annotation requirements. This is important because a limitation of this study is that training and test reports originated from a single institution. It is likely that the classifiers must be re-trained for other institutions due to report feature differences between institutions. Thus, although the pipeline training and implementation software are directly transferable between institutions, each institution would need to provide a labeled data set. Utilizing a HIL iterative approach could reduce the number of required training samples and therefore increase portability to other institutions. Interactive HIL ML concepts could potentially address another limitation of this study, namely our pipeline produces abnormality labels for anatomical regions (e.g. inner ear), rather than specific feature abnormalities (e.g. enlarged vestibular aqueduct). It is much more difficult to automate such classification primarily because it places significant burden on the domain experts to label a larger training set and to use detailed ontology concepts for annotation. An interactive HIL ML procedure could potentially be used to simplify the detailed annotation. Similar to reducing the overall training set size, in this approach the domain expert initially provides high-level concept labels that are used by the learning algorithm to suggest more detailed labels that the expert can accept or reject [22]. In this manner, granular labels are achieved without requiring the expert annotator to learn a complex ontology.

The presented work may be readily extended to clinical settings. As shown by the receiver operator characteristic results, the logistic regression model can be applied across all regions and is easily tuned to improve sensitivity (specificity) without severely impacting specificity (sensitivity). This may enable further applications of the described approach such as the use of NLP as a screening tool. For example, tuning to achieve high sensitivity may be used to reduce the need for physician review of predicted *normal* reports.

Our application of open source machine libraries and human-in-the-loop machine learning approaches on a novel pediatric audiologic data source further validates their potential and serves to demonstrate the generalizability of these methods. Additionally, while many biomedical research studies have considered ML and NLP approaches, their adoption in production biomedical software settings has been limited. This work provides evidence that such methods can be effectively utilized in production biomedical software environments.

## Conclusions

Our study was designed with the objective of enabling researchers to search the AudGenDB database for radiology reports that contain abnormalities in specific ear regions. The results indicate that the ML and NLP methods can achieve accuracy scores across the four regions (range 82–93 %) that approach physician expert performance as observed in previous studies [32–35]. We have implemented the classifier models in a web service that is utilized to generate labels for the radiology reports in AudGenDB. The labels are provided as a data field in the AudGenDB interface and can be used to filter reports for abnormalities in the four ear regions. To date, the web service has been used to label and enhance the search capability of over 16,000 radiologist reports in the AudGenDB database.

In summary, we developed an automated pipeline that labels radiologist text reports as *normal* or *abnormal* relative to four ear regions*.* Our results indicate that standard ML and NLP methods implemented in freely available software libraries in concert with HIL ML techniques can accurately identify abnormal regions noted in text reports for the otologic domain. This is encouraging in that it aligns with previous studies that have indicated the ability of such methods to accurately provide radiology report outcome labels and suggests general applicability of the approach.

## Abbreviations

AUC, area under the curve; AudGenDB, Audiological and Genetic Database; CHOP, The Children’s Hospital of Philadelphia; CT, computed tomography; EHR, electronic health record; FV, feature vector; G-SVM, Gaussian support vector machine; HIL, human in the loop; ML, machine learning; NLP, natural language processing; REST, representational state transfer; ROC, receiver operating characteristic; SVM, support vector machine.

## Additional file

Additional file 1: Keywords and ICD9 codes used for alternative document classification methods. (DOCX 131 kb)
