# Supplementary material for: Temporal bone radiology report classification using open source machine learning and natural langue processing libraries
Source: BMC Med Inform Decis Mak. 2016 Jun 6;16:65. doi: 10.1186/s12911-016-0306-3 (PMC4896018; doi:10.1186/s12911-016-0306-3)
Supplement: Additional file 1: — Keywords and ICD9 codes used for alternative document classification methods. (DOCX 131 kb) [file 12911_2016_306_MOESM1_ESM.docx]

# Supplementary Material

## Keyword search

The keywords used for each region in performing the keyword search baseline as described in the main text are provided here in Table 1.

Table 1: Keywords by region

| **Inner** | **Middle** | **Outer** | **Mastoid** |
| --- | --- | --- | --- |
| choclea | tympanic | external | mastoid |
| vestibular | ossicles | auditory canal | mastoids |
| vestibule | stapes |  |  |
| vestibules | incus |  |  |
| semicircular | malleus |  |  |
|  | scutum |  |  |

## ICD9 Codes

The International Classification of Diseases (ICD9) codes used for each region in performing the ICD9 based search as described in the main text are provided here in Table 2-5.

Table 2: ICD9 codes for inner ear

| **Code** | **Description** |
| --- | --- |
| 389.04 | CONDUCTIVE HEARING LOSS INNER EAR |
| 744.05 | CONGENITAL ANOMALIES OF INNER EAR |

Table 3: ICD9 codes for middle ear

| **Code** | **Description** |
| --- | --- |
| 160.1 | MALIGNANT NEOPLASM OF AUDITORY TUBE MIDDLE EAR AND MASTOID AIR CELLS |
| 212.0 | BENIGN NEOPLASM OF NASAL CAVITIES MIDDLE EAR AND ACCESSORY SINUSES |
| 382.00 | ACUTE SUPPURATIVE OTITIS MEDIA WITHOUT SPONTANEOUS RUPTURE OF EARDRUM |
| 382.01 | ACUTE SUPPURATIVE OTITIS MEDIA WITH SPONTANEOUS RUPTURE OF EARDRUM |
| 385.02 | TYMPANOSCLEROSIS INVOLVING TYMPANIC MEMBRANE AND EAR OSSICLES |
| 385.03 | TYMPANOSCLEROSIS INVOLVING TYMPANIC MEMBRANE EAR OSSICLES AND MIDDLE EAR |
| 385.10 | ADHESIVE MIDDLE EAR DISEASE UNSPECIFIED AS TO INVOLVEMENT |
| 385.19 | OTHER MIDDLE EAR ADHESIONS AND COMBINATIONS |
| 385.22 | IMPAIRED MOBILITY OF OTHER EAR OSSICLES |
| 385.23 | DISCONTINUITY OR DISLOCATION OF EAR OSSICLES |
| 385.24 | PARTIAL LOSS OR NECROSIS OF EAR OSSICLES |
| 385.32 | CHOLESTEATOMA OF MIDDLE EAR |
| 385.33 | CHOLESTEATOMA OF MIDDLE EAR AND MASTOID |
| 385.35 | DIFFUSE CHOLESTEATOSIS OF MIDDLE EAR AND MASTOID |
| 385.82 | CHOLESTERIN GRANULOMA OF MIDDLE EAR |
| 385.83 | RETAINED FOREIGN BODY OF MIDDLE EAR |
| 385.89 | OTHER DISORDERS OF MIDDLE EAR AND MASTOID |
| 385.9 | UNSPECIFIED DISORDER OF MIDDLE EAR AND MASTOID |
| 389.03 | CONDUCTIVE HEARING LOSS MIDDLE EAR |
| 744.03 | CONGENITAL ANOMALY OF MIDDLE EAR EXCEPT OSSICLES |
| 744.04 | CONGENITAL ANOMALIES OF EAR OSSICLES |

Table 4: ICD9 codes for outer ear

| **Code** | **Description** |
| --- | --- |
| 172.2 | MALIGNANT MELANOMA OF SKIN OF EAR AND EXTERNAL AUDITORY CANAL |
| 173.20 | UNSPECIFIED MALIGNANT NEOPLASM OF SKIN OF EAR AND EXTERNAL AUDITORY CANAL |
| 173.29 | OTHER SPECIFIED MALIGNANT NEOPLASM OF SKIN OF EAR AND EXTERNAL AUDITORY CANAL |
| 216.2 | BENIGN NEOPLASM OF EAR AND EXTERNAL AUDITORY CANAL |
| 380.13 | OTHER ACUTE INFECTIONS OF EXTERNAL EAR |
| 380.21 | CHOLESTEATOMA OF EXTERNAL EAR |
| 380.50 | ACQUIRED STENOSIS OF EXTERNAL EAR CANAL UNSPECIFIED AS TO CAUSE |
| 380.51 | ACQUIRED STENOSIS OF EXTERNAL EAR CANAL SECONDARY TO TRAUMA |
| 380.52 | ACQUIRED STENOSIS OF EXTERNAL EAR CANAL SECONDARY TO SURGERY |
| 380.53 | ACQUIRED STENOSIS OF EXTERNAL EAR CANAL SECONDARY TO INFLAMMATION |
| 380.81 | EXOSTOSIS OF EXTERNAL EAR CANAL |
| 380.89 | OTHER DISORDERS OF EXTERNAL EAR |
| 380.9 | UNSPECIFIED DISORDER OF EXTERNAL EAR |
| 389.01 | CONDUCTIVE HEARING LOSS EXTERNAL EAR |
| 744.01 | CONGENITAL ABSENCE OF EXTERNAL EAR |
| 744.02 | OTHER CONGENITAL ANOMALIES OF EXTERNAL EAR WITH IMPAIRMENT OF HEARING |

Table 5: ICD9 codes for mastoid region

| **Code** | Description |
| --- | --- |
| 160.1 | MALIGNANT NEOPLASM OF AUDITORY TUBE MIDDLE EAR AND MASTOID AIR CELLS |
| 385.33 | CHOLESTEATOMA OF MIDDLE EAR AND MASTOID |
| 385.35 | DIFFUSE CHOLESTEATOSIS OF MIDDLE EAR AND MASTOID |
| 385.89 | OTHER DISORDERS OF MIDDLE EAR AND MASTOID |
| 385.9 | UNSPECIFIED DISORDER OF MIDDLE EAR AND MASTOID |
